# Peripapillary Nerve Fiber Layer Thickness and Optic Nerve Head Parameters in Patients Recovered from COVID-19: A Longitudinal Study

**DOI:** 10.1155/2022/4643973

**Published:** 2022-03-29

**Authors:** Mojtaba Abrishami, Kiana Hassanpour, SeyedehMaryam Hosseini, Nasser Shoeibi, Mohammad Reza Ansari-Astaneh, Zahra Emamverdian, Bahareh Gharib, Nasibeh Amini, Majid Abrishami

**Affiliations:** ^1^Eye Research Center, Mashhad University of Medical Sciences, Mashhad, Iran; ^2^Ophthalmic Research Center, Research Institute for Ophthalmology and Vision Science, Shahid Beheshti University of Medical Sciences, Tehran, Iran

## Abstract

**Purpose:**

To quantify the longitudinal changes of the optic nerve head (ONH) parameters and peripapillary retinal nerve fiber layer (pRNFL) thickness in patients recovered from coronavirus disease 2019 (COVID-19) using spectral-domain optical coherence tomography (OCT) analysis.

**Materials and Methods:**

In an observational longitudinal study, in patients recovered from COVID-19, ONH OCT images were recorded at least two weeks after recovery from the systemic disease as the baseline and after one and three-month follow-up. Ganglion cell complex (GCC) analysis, ONH parameters, and pRNFL thickness changes were measured.

**Results:**

A total of 36 eyes from 18 recovered COVID-19 patients including eleven (61.2%) females were studied. The average age was 35.5 ± 7.5 years. The pRNFL thickness in the nasal lower sector significantly decreased from 77 ± 18 *μ*m in the first post-COVID-19 month to 74 ± 10 *μ*m in the third month (*P*=0.8). The average, superior, and inferior pRNFL thickness remained unchanged. The average cup volume significantly decreased from 0.27 ± 0.15 mm^3^ at baseline to 0.19 ± 0.15 mm^3^ in the third post-COVID-19 visit (*P*=0.028). In terms of ONH morphologies including rim, disc and cup to disc area, and the vertical and horizontal ratio, the changes were not significant over the 3-month study period. Focal loss volume and global loss volume values were not changed significantly.

**Conclusion:**

Localized defect in the nasal lower sector of pRNFL is observed in 3-month post-recovery from COVID-19. Larger studies with longer follow-ups are required to reveal the exact changes in ONH parameters.

## 1. Introduction

In the early days of the coronavirus disease 2019 (COVID-19) pandemic, ocular or neurologic evaluation seemed unnecessary. Severe acute respiratory syndrome coronavirus 2 (SARS-CoV-2), a member of the Coronaviridae family, was thought to lead to a respiratory illness, an infection in the lower respiratory tract that is transmitted especially through physical contact and respiratory droplets [1]. Later, coronavirus disease 2019 (COVID-19), besides its fatal pneumonia, was found to induce multiorgan failure and other systems involvement. Ophthalmic and neurologic involvements became among the common presentations of COVID-19 [2].

SARS-CoV-2 enters different cell types and triggers the innate immune response via its main receptor, angiotensin-converting enzyme (ACE) 2 [3]. The ACE2 receptors present in various types of cells in the body, including different cell types in the central nervous system [4]. Moreover, the ACE and ACE2 have been seen in the choroid and retina [5]. Hence, ophthalmic manifestations are inevitable. Results of primary reports on ocular manifestations of COVID-19 were focused on anterior segment presentations, such as conjunctivitis, conjunctival congestion, and chemosis [6]. Subsequently, although it is rare, decrease of retinal microvascular vessel density, other retinal vascular abnormalities, uveitis, and neuro-ophthalmic manifestations have been reported [7–12]. In our previous cross-sectional case-control study, we found a nonsignificant decrease in the pRNFL thickness of the recovered COVID-19 patient [13]. However, this study was cross-sectional and lack of follow-up of patients. Moreover, many other cross-sectional recent reports indicated defect in ONH and pRNFL, although the patients were not followed up in these studies as well [14, 15].

This longitudinal study aimed to evaluate ONH and pRNFL thickness in patients with a history of SARS-CoV-2 infection by SD-OCT and compare baseline measurements with one- and three-month follow-ups.

## 2. Materials and Methods

### 2.1. Study Participants

This longitudinal study was carried out at the Khatam Eye Hospital, the referral eye center of Mashhad University of Medical Sciences (MUMS), Mashhad, Iran. The inclusion criteria were a definite history of COVID-19 confirmed by the positive test result of a nasopharyngeal swab sample with real-time reverse transcription-polymerase chain reaction (RT-PCR) and a history of recovery from the systemic symptoms for at least one week. Detailed ocular and systemic histories of all the participants were collected. All the patients included in this study were the personnel of the Khatam Eye Hospital who had recovered from COVID-19. They volunteered to go through ophthalmological examination and ONH analysis in all three follow-up visits for the objectives of the present study.

The exclusion criteria were any history of refractive or intraocular surgery, history of diabetes mellitus, glaucoma, migraine, breastfeeding, current pregnancy, clinically apparent retinal disease, or autoimmune diseases. Those who had a history of hospitalization or systemic corticosteroid treatment for COVID-19 were not included. Furthermore, patients with a spherical refractive error greater than five diopters and a cylindrical refractive error of more than two diopters were also excluded. Any evidence of ocular media opacity preventing high-quality imaging or reduced OCT quality was also not included in the analysis. In addition, those with the best-corrected visual acuity less than 20/20 were also excluded from the protocol.

Personnel who were infected with COVID-19 and returned to their workplace two weeks after the completion of the symptomatic period were included in the study during the first week after their return. Patients were also imaged using the same machine one and three months after their return. All imaging was performed between 11 AM and 2 PM to avoid diurnal changes in vessel density and by one operator. Patients who did not complete the follow-up imaging were excluded from the study.

### 2.2. Image Acquisition and Analysis

All the SD-OCT scans were performed with the Optovue SD-OCT machine (RTVue XR Avanti, Optovue, Fremont, CA, USA; software version 2018.0.0.14). All measurements were performed using the automated default segmentation. We used the 3D disc image acquisition, ganglion cell complex (GCC) analysis, and ONH protocol of optic disc peripapillary evaluation to measure pRNFL thickness. GCC parameters were evaluated in this study, and they included the following: average thickness, thickness of the superior and inferior quadrant, the difference between the superior and inferior quadrant thickness (named intraeye), focal loss volume (FLV), and global loss volume (GLV).

All images were centered on the optic disc and displayed a signal strength of at least 50. All images in the study were carefully reviewed by the two retina specialists (MoA and SMH) to ensure their adequate quality, resolution, optic disc acquisition, and cup and rim detection. Moreover, the images with significant motion artifacts that interfered with evaluation were not included. ONH protocol evaluates pRNFL thicknesses in eight peripapillary segments and ONH cup, disc, and rim area analysis. Moreover, macula was also analyzed using GCC analysis to evaluate ganglion cell layer thickness and focal loss volume (FLV) and global loss volume (GLV) value changes. All subjects were examined using the same machine located in Khatam Eye Hospital, Mashhad, Iran. For each subject, both eyes were included.

### 2.3. Statistical Analysis

To present the data, descriptive statistics were used, including mean, median, standard deviation (SD), and range. The normal distribution of variables was tested using the Shapiro-Wilk test; and normality plots and homogeneity of variances were examined by Levene's test. To compare changes within the study subjects, generalized estimating equation analyses were performed. It should be mentioned that a *P* value of 0.05 was considered statistically significant. All statistical analyses were performed using the SPSS software for Windows (version 25).

### 2.4. Ethical Considerations

The study was performed based on the tenets of the Declaration of Helsinki. Accordingly, written informed consent was obtained from all participants before enrollment, and the study was ethically approved by the Regional Committee on Medical Ethics at Mashhad University of Medical Sciences (IR.MUMS.MEDICAL.REC.1399.402).

## 3. Results

A total of 36 eyes from 18 recovered COVID-19 patients were included. The average age ± SD of participants was 35.5 ± 7.5 (25–51) years. Eleven (61.2%) of the study participants were female. None of the cases was vaccinated as no vaccine was available in the period of the study in Iran.

While, the average, superior, and inferior pRNFL thickness remained unchanged over the study period, pRNFL thickness in the nasal lower sector significantly decreased from 77 ± 18 *μ*m in the first post-COVID-19 month to 74 ± 10 *μ*m in the third month (*P*=0.038). The thickness values remained unchanged in all other sectors (Table 1). Reduction in the lower nasal quadrant was observed in 23 eyes (63.8%) and 16 patients (88.8%) between the first and third visit. The range of thinning varied between −1 and −10.

**Figure 1:**
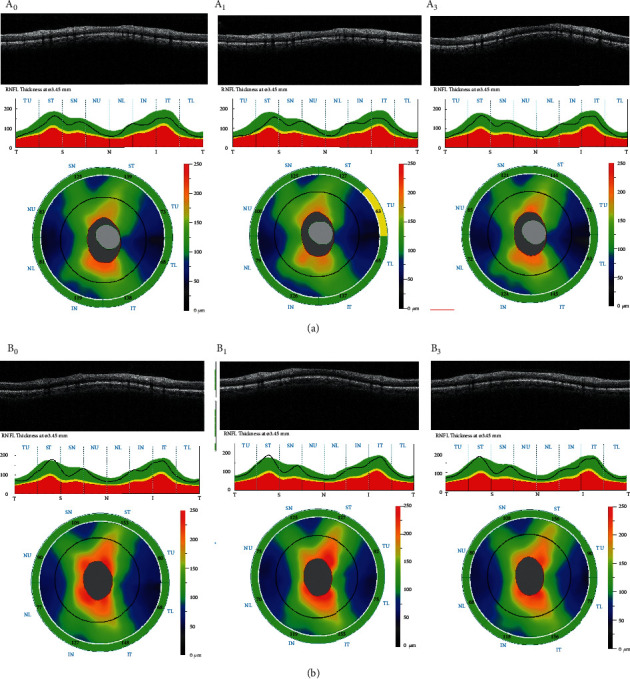
Representative spectral-domain optical coherence tomography images of the optic nerve head and peripapillary retinal nerve fiber layer thickness of two patients (a, b) at baseline and 1 and 3 months after COVID-19 recovery denoted by 0, 1, and 3.

Similarly, in terms of ONH morphologies including rim, disc, cup to disc area, and the vertical and horizontal ratio, the changes were not significant over the 3-month study period. However, the average cup volume significantly decreased from 0.27 ± 0.15 mm^3^ at baseline to 0.19 ± 0.15 mm^3^ in the third post-COVID-19 visit (*P*=0.028) (Table 1) (Figure 1).

Table 2 demonstrates the GCC parameters over the study period. The average GCC was 96 ± 5, 96 ± 6, and 97 ± 11 *μ*m in three respective post-COVID-19 visits (*P*=0.64). An increasing pattern was observed in FLV and GLV values. The average FLV was 0.8 ± 1.2 at baseline that increased to 0.86 ± 1.32 after 1 month and continued to rise to 1.02 ± 1.53 at month 3 (*P*=0.07). However, the increase did not reach a statistical significance level. A similar pattern was observed in GLV in a way that the average value at baseline (2.56 ± 2.9) reached 2.9 ± 3.37 and 3.06 ± 3.2 in the first and third post-recovery month (*P*=0.8) (Table 2).

## 4. Conclusions

The present study investigates the GCC analysis, pRNFL thickness, and ONH parameters in patients who recovered from COVID-19 over a 3-month follow-up period. Our results demonstrate that a localized decrease is observed in the nasal lower sector of pRNFL thickness 3 months after recovery from COVID-19. Furthermore, cup volume was significantly decreased over the study period while other pRNFL thickness and ONH parameters remained unchanged.

Two possible directions exist to explain the decrease in the nasal lower sector; first, the pRNFL thicknesses are increased upon the disease onset and the resolution of changes occurs during the recovery phases. Second, there is no change in ONH parameters and only focal changes are observed upon the disease onset and afterward. Since our COVID-19 cohort was evaluated after recovery and also there was no control group, it is difficult to understand which direction is more accurate. However, based on the few reports existing in the literature, pRNFL thickness is slightly increased in the acute phase of COVID-19 [13, 14]. Focal increase in the superior quadrant has also been reported compared to normal subjects [15]. Therefore, we speculate that the pRNFL thicknesses might be first increased and enjoy a fast resolution within the first weeks of the disease. The decrease in the nasal lower sector could be the consequence of further loss of nerve fibers and can be considered as a focal loss in pRNFL. To support our hypothesis, it is worth mentioning that the presence of focal defects after COVID-19 has been reported in another study. Ornek et al [16] demonstrated lower pRNFL thickness in the inferonasal quadrant in patients with COVID-19. The decrease in cup volume can be attributed to the first increase in the acute phase and resolution of changes in the recovery phase.

COVID-19 can affect the ONH through several possible mechanisms. The direct invasion of the SARS-CoV-2 virus is the first plausible mechanism. To date, the neuroinvasive behavior of the virus has been well demonstrated in both the central and peripheral nervous system [17, 18]. The presence of ACE2 receptors as the principal anchor of the virus on the retinal ganglion cells [19], the discovery of viral particles in the retina, and optic nerve of deceased patients by COVID-19 [20] are among the evidence of direct invasion. Furthermore, optic neuritis and neuroretinitis are commonly reported in association with various viral infections [21].

The effect of drugs commonly used in the treatment of COVID-19 can be another cause of pRNFL involvement. However, we could not find the reports of hydroxychloroquine [22], lopinavir/ritonavir, favipiravir, azithromycin, and NSAID impact on pRNFLs. Notwithstanding that none of the COVID-19 cohorts in the present study did receive corticosteroids or interferons for the treatment of the disease. Despite the lack of evidence, this mechanism should still be considered because it is possible that the pRNFL thickness is not evaluated thoroughly after receiving these drugs.

Another mechanism could be attributed to the role of inflammation and ischemia provoked by the disease. COVID-19 can damage the endothelial cells through the release of inflammatory markers and activated complement cascades [23, 24]. The impairment of endothelial cells in the vascular networks of ONH could cause an acute ischemic insult with the consequences of axonal stasis and nerve fibers swelling [25]. A recent report investigated the microvascular network of the ONH by OCT angiography and reported the microvascular impairment [26]. This observation could explain the acute edema observed in pRNFL and also consequent localized loss of pRNFL as a result of partially irreversible ischemia and eventual axonal atrophy [27].

The last plausible mechanism is the involvement of CNS in COVID-19. Neurological signs have been reported in 30 to 40 percent of COVID-19 patients [28]. Interestingly, in a study by Burgos-Blasco, post-COVID-19 patients with anosmia and ageusia had increased pRNFL and GCL compared with the patients without neurological signs [29]. The inflammation of CNS tissue can cause a subtle rise in ICP [30]. The increased pressure might be transmitted to the ONH through the subarachnoid space around the ONH similar to papilledema in exaggerated situations. Another interesting result of the present study is the increasing pattern observed in both GLV and FLV. In glaucoma patients, GLV and FLV equate to MD and PSD, respectively, and can be considered as sensitive markers of early glaucoma and advanced glaucoma diagnosis, respectively [31]. The increasing patterns in GLV and FLV might be related to the loss of retinal ganglion cells. However, it could also be attributed to the initial changes during the acute phase and the resolution of those changes within the recovery phase. Therefore, extra caution should be exerted to interpret these results, and further studies are required to understand the exact changes.

Various studies investigated macular or peripapillary OCT-A parameters in the follow-up of patients recovered from COVID-19 [32–34]. However, only a recently published longitudinal study is available on pRNFL thickness changes in severe COVID-19 patients, which showed significant decrease in pRNFL thickness in all quadrants in the recovery period compared with the active phase of the disease when the patients were hospitalized [15]. In our study, we evaluated the patients in their recovery period and found that the decrease is continual.

Several limitations of the present study are worth to be mentioned. First, the absence of control groups in study period unables us to compare the measured values with the eyes of the normal population. In our previous study, we used a cross-sectional case-control study protocol without follow-up of patients. Moreover, many other cross-sectional recent reports indicated defect in ONH and pRNFL, although the patients were not also followed up in these studies. In this study, we planned to evaluate the longitudinal changes, and it seems that using a control group may be redundant. However, it may be considered as a limitation to our study. Second, our small cohort of COVID-19 patients represents the mild spectrum of the disease. Therefore, the generalizability to all patients recovering from COVID-19 is reduced. Beside mild to moderate severity of the disease in our patients, our cohort of patients were relatively young and also otherwise healthy without any other systemic or ocular comorbidities. We believe that it can be considered as an advantage of our study, as if we find any change in this group of patient it may be attributable to COVID-19, by reducing power of other confounding factors. Changes may be worsened in older people or patients with systemic comorbidity. Third, both eyes of each patient were included, which may interfere with the analysis. Fourth, anosmia which can be considered a neurotropic characteristic of SARS-CoV-2 is not evaluated in the present study. Fifth, patients cannot be assured that they do not have asymptomatic COVID-19 due to the lack of a PCR test and laboratory characteristics performed at the follow-up visits. As it is not a routine procedure to apply RT-PCR test in follow-ups, we did not perform it, and it could be considered as a limitation.

In conclusion, the localized defect in the nasal lower sector of pRNFL is observed in 3 months postrecovery from COVID-19. The resolution of higher pRNFL thickness proposed by other investigations may be fast. The resolution could be followed by a localized defect that implies partial recovery from the acute insult. Larger studies with longer follow-ups are required to reveal the exact changes in ONH parameters.

## Figures and Tables

**Table 1:** Peripapillary optical coherence tomography parameters over the study period.

		First visit	1^st^ month	3^rd^ month	*P* value^*∗*^	
Average RNFL	Mean ± SD	100 ± 9	100 ± 10	100 ± 9	0.89	
Superior RNFL	Mean ± SD	102 ± 9	102 ± 10	102 ± 8	0.78	
Inferior RNFL	Mean ± SD	99 ± 11	98 ± 11	98 ± 10	0.82	
Cup to disc area	Mean ± SD	0.36 ± 0.16	0.35 ± 0.16	0.36 ± 0.15	0.99	
Cup to disc vertical ratio	Mean ± SD	0.53 ± 0.19	0.52 ± 0.17	0.53 ± 0.16	0.80	
Cup to disc horizontal ratio	Mean ± SD	0.64 ± 0.21	0.64 ± 0.2	0.65 ± 0.19	0.79	
Rim area	Mean ± SD	1.23 ± 0.31	1.29 ± 0.25	1.29 ± 0.23	0.64	
Disc area	Mean ± SD	1.97 ± 0.44	2.02 ± 0.31	2.03 ± 0.28	0.96	
Cup volume	Mean ± SD	0.22 ± 0.155	0.20 ± 0.15	0.19 ± 0.15	0.001	1, 3 (0.028)
Superior temporal	Mean ± SD	140 ± 14	137 ± 16	139 ± 12	0.27	
Superior nasal	Mean ± SD	109 ± 15	110 ± 17	109 ± 13	0.85	
Nasal upper	Mean ± SD	81 ± 12	81 ± 16	79 ± 12	0.45	
Nasal lower	Mean ± SD	76 ± 10	77 ± 18	74 ± 10	0.013	2, 3 (0.038)
Inferior nasal	Mean ± SD	113 ± 20	114 ± 20	114 ± 18	0.99	
Inferior temporal	Mean ± SD	138 ± 15	135 ± 17	139 ± 14	0.44	
Temporal lower	Mean ± SD	67 ± 8	66 ± 8	67 ± 8	0.62	
Temporal upper	Mean ± SD	79 ± 9	78 ± 11	79 ± 9	0.85	

^*∗*^Based on generalized estimating equation. RNFL: retinal nerve fiber layer. SD: standard deviation.

**Table 2:** Ganglion cell complex analysis over the study period.

		First visit	1^st^ month	3^rd^ month	*P* value^*∗*^
Average GCC (*μ*m)	Mean ± SD	96 ± 5	96 ± 6	97 ± 11	0.64
Superior GCC (*μ*m)	Mean ± SD	95 ± 4	95 ± 5	96 ± 9	0.47
Inferior GCC (*μ*m)	Mean ± SD	96 ± 6	96 ± 7	98 ± 14	0.87
FLV (%)	Mean ± SD	0.8 ± 1.22	0.86 ± 1.32	1.02 ± 1.53	0.07
GLV (%)	Mean ± SD	2.56 ± 2.9	2.90 ± 3.37	3.06 ± 3.2	0.8

^*∗*^Based on generalized estimating equation; GCC: ganglion cell complex; FLV: focal loss volume; GLV global loss volume.

## Data Availability

The datasets generated and analyzed during the current study are available from the corresponding author on reasonable request.

## References

[1] Palmieri L., Palmer K., Lo Noce C. (2021). Differences in the clinical characteristics of COVID-19 patients who died in hospital during different phases of the pandemic: national data from Italy. *Aging-Clinical & Experimental Research*.

[2] de Oliveira F. A. A., Palmeira D. C. C., Rocha-Filho P. A. S. (2020). Headache and pleocytosis in CSF associated with COVID-19: case report. *Neurological Sciences*.

[3] Gheblawi M., Wang K., Viveiros A. (2020). Angiotensin-converting enzyme 2: SARS-CoV-2 receptor and regulator of the renin-angiotensin system. *Circulation Research*.

[4] Zhou Z., Kang H., Li S., Zhao X. (2020). Understanding the neurotropic characteristics of SARS-CoV-2: from neurological manifestations of COVID-19 to potential neurotropic mechanisms. *Journal of Neurology*.

[5] Choudhary R., Kapoor M. S., Singh A., Bodakhe S. H. (2017). Therapeutic targets of renin-angiotensin system in ocular disorders. *Journal of Current Ophthalmology*.

[6] Abrishami M., Tohidinezhad F., Daneshvar R. (2020). Ocular manifestations of hospitalized patients with COVID-19 in northeast of Iran. *Ocular Immunology and Inflammation*.

[7] Abrishami M., Emamverdian Z., Shoeibi N. (2021). Optical coherence tomography angiography analysis of the retina in patients recovered from COVID-19: a case-control study. *Canadian Journal of Ophthalmology*.

[8] Gascon P., Briantais A., Bertrand E. (2020). Covid-19-associated retinopathy: a case report. *Ocular Immunology and Inflammation*.

[9] Ghodsieh Z., Azimi A., Ali A. (2021). Acute macular neuroretinopathy in a patient with acute myeloid leukemia and deceased by COVID-19: a case report. *Journal of Ophthalmic Inflammation and Infection*.

[10] Hosseini S. M., Abrishami M., Zamani G. (2021). Acute bilateral neuroretinitis and panuveitis in a patient with coronavirus disease 2019: a case report. *Ocular Immunology and Inflammation*.

[11] Ortiz-Seller A., Martínez Costa L., Hernández-Pons A., Valls Pascual E., Solves Alemany A., Albert-Fort M. (2020). Ophthalmic and neuro-ophthalmic manifestations of coronavirus disease 2019 (COVID-19). *Ocular Immunology and Inflammation*.

[12] Virgo J., Mohamed M. (2020). Paracentral acute middle maculopathy and acute macular neuroretinopathy following SARS-CoV-2 infection. *Eye*.

[13] Abrishami M., Daneshvar R., Emamverdian Z., Tohidinezhad F., Eslami S. (2021). Optic nerve head parameters and peripapillary retinal nerve fiber layer thickness in patients with coronavirus disease 2019. *Ocular Immunology and Inflammation*.

[14] Burgos‐Blasco B., Güemes‐Villahoz N., Donate‐Lopez J., Vidal‐Villegas B., García‐Feijóo J. (2021). Optic nerve analysis in COVID‐19 patients. *Journal of Medical Virology*.

[15] Mavi Yildiz A., Ucan Gunduz G., Yalcinbayir O., Acet Ozturk N. A., Avci R., Coskun F. (2021). SD-OCT assessment of macular and optic nerve alterations in patients recovered from COVID-19. *Canadian Journal of Ophthalmology*.

[16] Örnek K., Temel E., Aşıkgarip N., Kocamış Ö. (2021). Localized retinal nerve fiber layer defect in patients with COVID-19. *Arquivos Brasileiros de Oftalmologia*.

[17] Lechien J. R., Chiesa-Estomba C. M., De Siati D. R. (2020). Olfactory and gustatory dysfunctions as a clinical presentation of mild-to-moderate forms of the coronavirus disease (COVID-19): a multicenter European study. *European Archives of Oto-Rhino-Laryngology*.

[18] Steardo L., Steardo L., Zorec R., Verkhratsky A. (2020). Neuroinfection may contribute to pathophysiology and clinical manifestations of COVID‐19. *Acta Physiologica*.

[19] Mahalingam R., Dharmalingam P., Santhanam A. (2021). Single‐cell RNA sequencing analysis of SARS‐CoV‐2 entry receptors in human organoids. *Journal of Cellular Physiology*.

[20] Casagrande M., Fitzek A., Spitzer M. (2021). Detection of SARS-CoV-2 genomic and subgenomic RNA in retina and optic nerve of patients with COVID-19. *British Journal of Ophthalmology*.

[21] Kahloun R., Abroug N., Ksiaa I. (2015). Infectious optic neuropathies: a clinical update. *Eye and Brain*.

[22] Lee E. J., Kim S. J., Han J. C. (2018). Peripapillary retinal nerve fiber layer thicknesses did not change in long-term hydroxychloroquine users. *Korean Journal of Ophthalmology*.

[23] Marchetti M. (2020). COVID-19-driven endothelial damage: complement, HIF-1, and ABL2 are potential pathways of damage and targets for cure. *Annals of Hematology*.

[24] Zhang J., Tecson K. M., McCullough P. A. (2020). Endothelial dysfunction contributes to COVID-19-associated vascular inflammation and coagulopathy. *Reviews in Cardiovascular Medicine*.

[25] Nukada H., Dyck P. J. (1987). Acute ischemia causes axonal stasis, swelling, attenuation, and secondary demyelination. *Annals of Neurology*.

[26] Savastano A., Crincoli E., Savastano M. C. (2020). Peripapillary retinal vascular involvement in early post-COVID-19 patients. *Journal of Clinical Medicine*.

[27] Johnson M. A., Miller N. R., Nolan T., Bernstein S. L. (2016). Peripapillary retinal nerve fiber layer swelling predicts peripapillary atrophy in a primate model of nonarteritic anterior ischemic optic neuropathy. *Investigative Opthalmology & Visual Science*.

[28] Li H., Xue Q., Xu X. (2020). Involvement of the nervous system in SARS-CoV-2 infection. *Neurotoxicity Research*.

[29] Burgos-Blasco B., Güemes-Villahoz N., Vidal-Villegas B. (2021). Optic nerve and macular optical coherence tomography in recovered COVID-19 patients. *European Journal of Ophthalmology*.

[30] Silva M. T. T., Lima M. A., Torezani G. (2020). Isolated intracranial hypertension associated with COVID-19. *Cephalalgia*.

[31] Arintawati P., Sone T., Akita T., Tanaka J., Kiuchi Y. (2013). The applicability of ganglion cell complex parameters determined from SD-OCT images to detect glaucomatous eyes. *Journal of Glaucoma*.

[32] Cennamo G., Reibaldi M., Montorio D., D’Andrea L., Fallico M., Triassi M. (2021). Optical coherence tomography angiography features in post-COVID-19 pneumonia patients: a pilot study. *American Journal of Ophthalmology*.

[33] Burgos-Blasco B., Güemes-Villahoz N., Vidal-Villegas B. (2021). Optic nerve head vessel density assessment in recovered COVID-19 patients: a prospective study using optical coherence tomography angiography. *Journal of Glaucoma*.

[34] Abrishami M., Hassanpour K., Hosseini S. M. (2021). Macular vessel density reduction in patients recovered from COVID-19: a longitudinal optical coherence tomography angiography study. *Graefes Archive for Clinical and Experimental Ophthalmology*.

